# The Flow Rate in Patients With Low-Gradient Aortic Stenosis

**DOI:** 10.7759/cureus.60776

**Published:** 2024-05-21

**Authors:** Marina Leitman, Mohameed Daoud, Vladimir Tyomkin, Shmuel Fuchs

**Affiliations:** 1 Sackler School of Medicine, Tel Aviv University, Tel Aviv, ISR; 2 Cardiology, Shamir Medical Center, Zerifin, ISR

**Keywords:** low gradient, flow status, stroke volume index, predictor of mortality, aortic stenosis, flow rate

## Abstract

Purpose: The decision to assess the severity and determine the ideal timing of intervention for low-gradient aortic stenosis poses a greater challenge. Recently, a novel method for determining the flow status of patients with aortic stenosis has been introduced, utilizing flow rate measurements. In this study, we investigated whether the flow status of patients with low-gradient aortic stenosis is linked to mortality within a three-year timeframe.

Methods: Twenty-nine patients diagnosed with low-gradient aortic stenosis and valve area ≤ 1 cm were identified during 2010-2015. Each patient's flow rate across the aortic valve was computed, and the study scrutinized echocardiographic parameters to ascertain their correlation with mortality over a three-year timeframe.

Results: We observed that among patients with low-gradient aortic stenosis and a valve area of ≤1 cm, a decreased flow rate across the aortic valve emerged as an independent predictor of mortality. A flow rate < 210 ml/s was linked with a three-year mortality rate of 66.7%, whereas a low stroke volume index < 35 ml/m² did not show an association with three-year mortality. This observation might be attributed to the smaller body sizes prevalent among these older patients, particularly females, which could influence the calculation of the stroke volume index.

Conclusion: In older patients with low-gradient aortic stenosis, the flow rate can better reflect flow status than the stroke volume index, and it also suggests a prognostic significance in predicting mortality. Additional studies are warranted to validate these findings across broader patient populations and to assess the potential efficacy of early intervention strategies in this particular patient cohort.

## Introduction

Based on a large body of evidence, recommendations exist regarding the optimal timing for intervention in cases of severe high-gradient aortic stenosis. Severe high-gradient aortic stenosis is defined, per the latest guidelines, as having a mean pressure gradient of 40 mmHg or greater and a calculated aortic valve area of ≤1 cm² [1]. Determining the severity and optimal timing of intervention for low-gradient severe aortic stenosis is a more challenging decision. If the aortic valve area is less than 1 cm² and the mean gradient is below 40 mmHg (low-gradient severe aortic stenosis), the flow status needs to be assessed. To assess the flow status, the stroke volume index is computed. If the stroke volume index is 35 ml/m² or greater, it is unlikely that low flow is present, and the stenosis is more likely to be moderate in severity. Additionally, the calculation of the aortic valve area may be inaccurate. Patients with low-gradient severe aortic stenosis and a stroke volume index ≥ 35 ml/m² can be managed similarly to those with moderate aortic stenosis. If the stroke volume index is less than 35 ml/m², low-flow low-gradient severe aortic stenosis is diagnosed.

Low-dose dobutamine stress echocardiography is recommended in patients with reduced ejection fraction to distinguish between true severe aortic stenosis and moderate aortic stenosis and assess contractile reserve. Low-dose dobutamine stress echocardiography may not be feasible or safe in all patients. In some patients, the aortic valve area may remain narrow despite an insufficient increase in the pressure gradient on the aortic valve (less than 40 mmHg). While the projected aortic valve area can be calculated, it is not yet part of the guideline recommendations. In cases of low-flow low-gradient severe aortic stenosis with preserved left ventricular function (ejection fraction ≥ 50%) and in cases where low-dose dobutamine stress echocardiography is not feasible, the use of computerized tomography calcium score may be helpful.

According to a report by Namasivayam et al. [2], the aortic valve area may not always be a reliable predictor of mortality in patients with aortic stenosis. According to the given statement, in patients with a flow rate through the aortic valve > 242 ml/s, an aortic valve area ≤ 1 cm² was found to be a strong predictor of mortality. However, in patients with a flow rate through the aortic valve ≤ 242 ml/s, an aortic valve area ≤ 1 cm² was not found to be a predictor of mortality. This suggests that the predictive value of aortic valve area for mortality may depend on the flow rate through the aortic valve, and other factors may be more important in patients with lower flow rates. In this study, a recommended optimal flow rate threshold of 210 ml/s was identified. When the mean pressure gradient is less than 40 mmHg and the flow rate is below 210 ml/s, an aortic valve area of 1 cm² or less does not have prognostic significance [2]. Vamvakidou et al.'s study found that a flow rate of less than 200 ml/s was an independent predictor of mortality in patients with low-gradient severe aortic stenosis who underwent aortic valve replacement [3]. This study examined the impact of flow rate low-gradient aortic stenosis patients with a valve area of 1 cm² or less.

## Materials and methods

The echocardiography database of Shamir Medical Center was used to identify patients who were diagnosed with at least moderate aortic stenosis between 2010 and 2015. Patients who had more than moderate mitral and aortic regurgitation were excluded. We also excluded patients who were lost to follow-up. We analyzed all patients for hemodynamic echocardiography findings as well as demographic and clinical data according to the hospital files. Echocardiography examinations were retrieved from digital archives. Offline retrospective echocardiography analysis included a revision of conventional echocardiography with re-calculation of aortic valve area, stroke volume index, left ventricular mass index, and calculation of flow rate through the aortic valve.

Patients with low-gradient aortic stenosis and valve area ≤ 1 cm² were identified. In these patients, the flow rate was calculated retrospectively. To address the issue that ejection time is not routinely measured in echocardiography studies for patients with aortic stenosis, we used the formula proposed by Namasivayam et al. [2] to calculate the flow rate in our revised retrospective echocardiography studies:

$Q= AVA \times V_{m} = AVA \times \frac{[MG\ +\sqrt{(MG^2\ +\ 32\ \times\ MG\ \times\ V_{p}^{2})]}}{[16\ \times\ V_p]}$

where Q is the flow rate through the aortic valve, AVA is an area of the aortic valve, V_m_ is the mean velocity through the aortic valve, MG is the mean gradient on the aortic valve, and V_p_ is the peak velocity through the aortic valve [2].

All echocardiography exams in our hospital were performed using the echocardiography system Vivid E9 (General Electric; Horten, Norway) with a standard transducer of 1.7-4 Hz. Comprehensive transthoracic echocardiography examinations were performed according to the standard recommendations on chamber quantification [4].

The aortic valve area was determined via the continuity equation, employing the ratio of velocity time integrals [5]. Stroke volume index was computed using the Doppler method, adhering to standard guideline recommendations [5], by multiplying the left ventricular outflow tract area, as calculated, with the left ventricular velocity time integral and then adjusting for patient body surface area (BSA). BSA was calculated according to Mosteller [6].

To establish a comparative reference, this study juxtaposed the echocardiographic hemodynamics and demographic profiles of patients diagnosed with low-gradient significant aortic stenosis against those of patients diagnosed with moderate aortic stenosis.

This retrospective study aimed to investigate the three-year mortality in patients with aortic stenosis and valve area ≤ 1 cm², with the endpoint of the study being the aforementioned mortality rate. The outcome of the study was assessed based on digital hospital data, with a follow-up period of three years.

Statistical methods

We expressed continuous data as means ± standard deviations (SD). We used a paired t-test and a two-sample student's t-test to compare means as appropriate. When variances were unequal, we used Welch's t-test. Statistical analysis was performed using a 95% confidence interval, and statistical significance was determined using a threshold of p < 0.05. The data that fell under categorical variables were presented as numerical values and percentages. To conduct univariate analysis, the chi-square/Fisher's exact test was utilized as appropriate. The normal distribution of all variances was tested through the Kolmogorov-Smirnov test. For multivariate analysis, an ANOVA model was employed using IBM SPSS Statistics for Windows, version 28.0 (IBM Corp., Armonk, NY).

Ethical approval was not required for this study dealing with patients' files as it entailed retrospective and anonymous data collection that did not impact clinical diagnosis or patient management.

## Results

**Figure 1:**
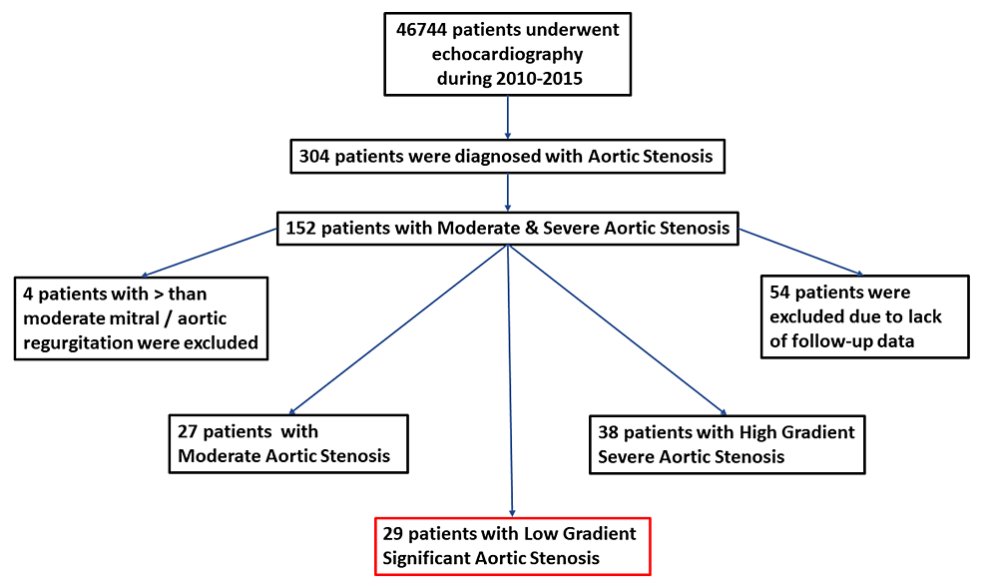
Flow chart of the patients with moderate and severe aortic stenosis

A total of 46744 patients underwent an echocardiography examination in the Shamir Medical Center in 2010-2015 (Figure 1).

Of them, 304 were diagnosed with aortic stenosis, and in 152 patients, aortic stenosis was moderate or severe. Fifty-four patients were excluded due to the lack of follow-up. Four patients were excluded due to concomitant significant mitral and/or aortic regurgitation. Thirty-eight patients had high-gradient severe aortic stenosis, 24 patients had moderate aortic stenosis, and 29 patients had low-gradient aortic stenosis (mean gradient < 40 mmHg, and valve area ≤ 1 cm). This group of patients with low-gradient significant aortic stenosis was analyzed in this study. General clinical and demographic data are presented in Table 1.

**Table 1:** General patients' characteristics BSA: Body surface area; HPTN: Hypertension; DM: Diabetes mellitus; CAD: Coronary artery disease; MI: Myocardial infarction; CRF; Chronic renal failure; CHF: Congestive heart failure; PVD: Peripheral vascular disease; ACEi: Angiotensin-converting enzyme inhibitors; ARB: Aldosterone receptor blockers; Ca blockers: Calcium channel blockers; Antiplatelet Rx: Antiplatelet medications.

Variables	Value
Number of patients	29
Age, years	81.6 ± 7.1
Female, n (%)	20 (69%)
Male, n (%)	9 (31%)
Weight, kg	66.3 ± 13.7
Height, cm	160.3 ± 8.5
BSA, m²	1.7 ± 0.2
HPTN, n (%)	26 (89.7%)
DM, n (%)	15 (51.7%)
CAD, n (%)	18 (62.1%)
MI, n (%)	14 (48.3%)
CRF, n (%)	15 (51.7%)
Smoker, n (%)	5 (17.2%)
Atrial fibrillation, n (%)	17 (58.6%)
CHF, n (%)	19 (65.5%)
PVD, n (%)	15 (51.7%)
Hyperlipidemia, n (%)	17 (58.6%)
Beta-blockers, n (%)	20 (69%)
ACEi, n (%)	8 (27.6%)
ARB, n (%)	4 (13.8%)
Ca blockers, n (%)	9 (31.0%)
Antiplatelet Rx, n (%)	17 (58.6%)
Statins, n (%)	16 (55.2%)
Anticoagulation, n (%)	14 (48.3%)

About 31% were males, nearly 90% were hypertensive, 65.5% had congestive heart failure, 51.7% had renal failure, diabetes, peripheral vascular disease, and 62.1% had coronary artery disease. Echocardiography analysis is presented in Table 2.

**Table 2:** Echocardiographic patients' characteristics EF: Ejection fraction; SVi: Stroke volume index; Q: Flow rate; PG: Peak gradient; MG: Mean gradient; AVA: Aortic valve area; LVEDD: Left ventricular end-diastolic diameter; LVESD: Left ventricular end-systolic diameter; LAD: Left atrial diameter; LAA: Left atrial area; LVMi: Left ventricular mass index; PAP: Pulmonary artery pressure.

Variables	Value, mean ± SD
EF, %	51.7 ± 11
Svi, ml/m²	39.7 ± 11.2
Q, ml/s	212.8 ± 47.8
PG, mmHg	45.8.6 ± 12.5
MG, mmHg	28.0 ± 8
AVA, cm²	0.88 ± 0.15
LVEDD, cm	4.7 ± 0.7
LVESD, cm	2.8 ± 0.7
LAD, cm	4.4 ± 0.8
LAA, cm²	27.1 ± 6.7
LVMi, g/m²	118.9 ± 25.7
PAP, mmHg	51.6 ± 14.6

The mean EF in these patients was found to be preserved, with a value of 51.7 ± 11%, and the stroke volume index was 39.7 ± 11.2 ml/m². Thirteen patients were found to have an aortic valve area of 1 cm², which was consistent with a diagnosis of moderate aortic stenosis according to the 2012 guidelines and were not referred for intervention. Two patients refused transcatheter aortic valve implantation (TAVI), eight patients had unequivocal symptoms and were not referred for intervention, and six patients were deferred from TAVI due to comorbidities in medical decisions. The mean flow rate across the aortic valve was 212.8 ± 47.8 ml/s. Thirteen patients (44.8%) in this group died during the three years of follow-up. Multivariate analysis demonstrated that the flow rate was a stronger predictor of three-year mortality compared to ejection fraction (p = 0.007 and p < 0.03, respectively), while age and stroke volume index did not show significant associations with mortality (p = 0.17 and p = 0.11, respectively). A flow rate of less than 210 ml/s was predictive of mortality in patients with low-gradient moderate-severe aortic stenosis as presented in Table 3.

**Table 3:** A flow rate of <210 ml/s for prediction of three-year mortality in patients with low-gradient aortic stenosis and a valve area of ≤1 cm² PPV: Positive predictive value; NPV: Negative predictive value; FPR: False positive ratio; FNR: False negative rate.

PPV	NPV	Sensitivity	Specificity	FPR	FNR	Accuracy
66.7%	78.6%	76.9%	68.8%	31.3%	23.1%	72.4%

Among the cohort, 10 patients exhibited a reduced ejection fraction (<50%), while 19 patients demonstrated a preserved ejection fraction (≥50%). Notably, there was no significant disparity in mortality rates between the two groups over three years: six (60%) versus seven (36.8%), with a p-value of 0.84 (nonsignificant).

**Figure 2:**
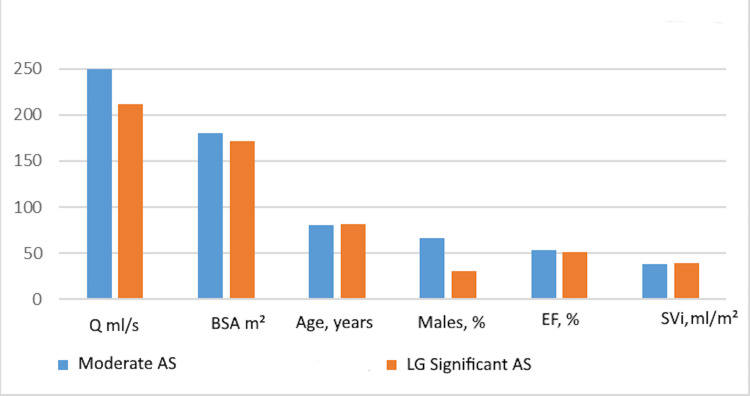
Hemodynamic and demographic characteristics of patients with low-gradient significant AS versus moderate AS AS: Aortic stenosis; LG: Low gradient; Q: Flow rate through the aortic valve; BSA: Body surface area; EF: Ejection fraction; Svi: Stroke volume index.

For reference, the hemodynamic and demographic characteristics of the patients with low-gradient significant aortic stenosis were compared with patients who had moderate aortic stenosis. Patients with moderate aortic stenosis were younger and had higher BSA, higher height, and higher flow rates through the aortic valve. There was no significant difference between the two groups regarding stroke volume and ejection fraction (Table 4, Figure 2).

**Table 4:** Hemodynamic and demographic characteristics of patients with low-gradient significant aortic stenosis versus moderate aortic stenosis AS: Aortic stenosis; LG: Low gradient; Svi: Stroke volume index; Q: Flow rate; EF: Ejection fraction; BSA: Body surface area.

Variables	Males, %	Age, years	Svi, ml/m²	Q, ml/s	EF, %	Weight, kg	Height, cm	BSA, m²
LG significant AS	31.0	81.6 ± 7.1	39.7 ± 11.2	212.8 ± 47.8	51.7 ± 11	66.3 ± 13.7	160.3 ± 8.5	1.71 ± 0.2
Moderate AS	66.7	80.3 ± 8	38.5 ± 10.3	249.3 ± 42.1	53.6 ± 12.3	72 ± 12.5	165.5 ± 6.7	1.80 ± 0.2
p-value	0.71	0.003	0.69	0.004	0.55	0.11	<0.02	<0.04

**Figure 3:**
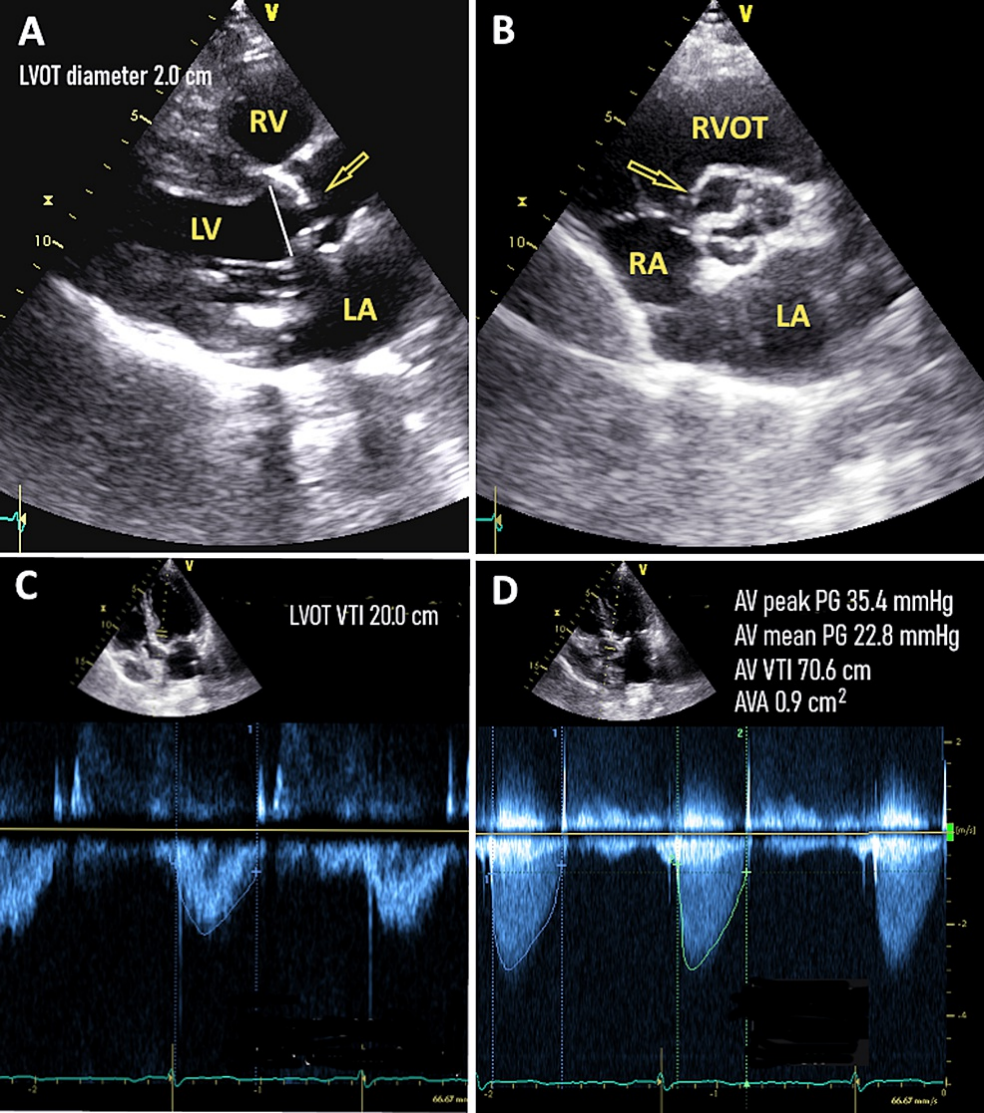
An example of the echocardiographic assessment of a 90-year-old lady with preserved left ventricular function and low-gradient severe aortic stenosis (A) Parasternal long axis view. A calcified aortic valve with a limited opening is seen (arrow). LVOT diameter was measured at 2.0 cm. (B) Parasternal short axis view. A calcified aortic valve with a limited opening is shown (arrow). (C) A pulsed wave Doppler through the left ventricle outflow tract at the five-chamber apical view is seen. (D) Continuous wave Doppler through the aortic valve at the five-chamber apical view is shown. The echocardiography examination of this patient is consistent with low-gradient severe aortic stenosis. Her weight was 71 kg, her height was 162 cm, and her BSA was calculated at 1.79 m². The stroke volume index was 35.3 ml/m² (borderline). The flow rate through the aortic valve was 196.8 ml/s (low). LV: Left ventricle; RV: Right ventricle; LA: Left atrium; RA: Right atrium; LVOT: Left ventricle outflow tract; RVOT: Right ventricle outflow tract; VTI: Velocity time integral; AVA: Aortic valve area; AV peak PG: Aortic valve peak pressure gradient; AV mean PG: Aortic valve mean pressure gradient.

An example of an echocardiography examination of an elderly female with low-gradient severe aortic stenosis, preserved left ventricular function, borderline non-conclusive stroke volume index, and low flow rate is demonstrated in Figure 3.

## Discussion

In our study, we found that among patients with significant aortic stenosis and low gradient (defined as aortic valve area ≤ 1 cm²), a reduced flow rate below 210 ml/s through the aortic valve was linked to mortality. However, we did not observe a similar correlation with mortality for a low stroke volume index (<35 ml/m²). The study population consisted of elderly patients, with 90% of them being female. These patients exhibited slightly lower body weight and height, resulting in a calculated BSA lower than that of younger patients. In elderly populations, stroke volume index adjusted to BSA may be a less reliable parameter for determining flow status in patients with aortic stenosis. As BSA tends to be lower in elderly patients compared to younger patients, the same stroke volume measurement may suggest a low-flow state in younger individuals but might be considered normal in the elderly. Aging is typically associated with a decline in muscle mass, particularly after the age of 60 [7]. Gender differences can also influence measurements of the aortic valve area, often resulting in an overestimation of the severity of aortic valve stenosis in females and individuals with smaller body sizes [8].

Accurate assessment of myocardial contractility is crucial for prognosis in patients with aortic stenosis. The guidelines for valvular heart disease have established a cutoff of 50% for ejection fraction as an indication for valve replacement in asymptomatic patients with severe aortic stenosis [1]. Previous studies have demonstrated that ejection fraction is a powerful predictor of outcome in both symptomatic and asymptomatic patients with severe high-gradient aortic stenosis with the best outcomes observed in those with an EF ≥ 70%, and a gradual reduction in survival as EF decreases to <50% [9,10]. Patients with normal flow aortic stenosis who underwent valve replacement had better survival compared to those with low-flow aortic stenosis, which was determined by stroke volume index [10-12].

A cutoff value of 35 ml/m^2^ for the stroke volume index has been suggested as a definition for low-flow aortic stenosis [13]. Patients who meet this criterion are typically female, exhibit a mean gradient across the aortic valve of less than 40 mmHg, and have preserved ejection fraction. Numerous studies aim to determine the optimal cutoff value for stroke volume index in predicting mortality among patients with low-gradient aortic stenosis [13-16]. Interestingly, paradoxical findings emerge regarding the stroke volume index as a predictor of heightened mortality in patients with low-gradient aortic stenosis and preserved or reduced ejection fraction. Notably, distinct cutoff values were proposed: <30 ml/m² for preserved ejection fraction and <35 ml/m² for reduced ejection fraction [16].

Last year's flow rate through the aortic valve has emerged as an alternative and complementary criterion for assessing flow status. Several studies have reported a threshold for a low-flow state significant for prognosis, ranging between 200 and 210 ml/s [2,3,17]. In a study by Alexandru et al. [18], flow rate demonstrated a stronger association with aortic valve area than stroke volume index.

Flow rate uses uncorrected stroke volume with respect to BSA but reflects aortic valve resistance [17]. Flow rate depends on transvalvular flow, which is the basic determinant of pressure gradient [17]. Transvalvular flow is contingent upon both preload and afterload dynamics. In clinical practice, patients exhibiting low flow and low-gradient characteristics often comprise elderly females with a reduced left ventricular cavity size [19]. Influential factors such as hypertension, diuretic therapy, and medications targeting afterload reduction via vasodilation can exert notable effects on flow rate.

Low flow through the aortic valve in patients with aortic stenosis can indicate intrinsic left ventricular dysfunction, even in the presence of preserved ejection fraction and stroke volume index. The severity of extra-valvular cardiac damage in patients with severe aortic stenosis was independently linked to increased mortality following aortic valve replacement [20].

The severity of aortic stenosis is not solely determined by the valve area or gradient but also by considering the patient's symptoms, clinical presentation, and overall health status. Many patients with moderate and severe aortic stenosis may not report any symptoms, and a delay in seeking medical attention when symptoms do arise is common. Therefore, routine monitoring and assessment of patients with aortic stenosis are crucial for early detection and intervention [21,22]. During each echocardiography examination, it is essential to evaluate different flow indices, including stroke volume index and flow rate to improve clinical management and decision-making processes effectively [23].

This study is limited by its retrospective design, relying on data collected from medical records rather than from a prospective, randomized trial. Moreover, the relatively small sample size may restrict the generalizability of the findings to broader patient populations. Furthermore, the study's focus solely on patients from a single center may further constrain the applicability of the results to other clinical settings.

## Conclusions

To summarize, the assessment of stroke volume index in older patients with aortic stenosis reveals its limitations as a sole indicator of flow status, primarily due to its dependence on BSA. Recognizing this, the consideration of flow rate emerges as a promising alternative and complementary criterion for evaluating flow status in this patient population. In the unique realm of aortic stenosis and low-gradient among older patients, our study, albeit based on a small patient population, uncovers a pivotal flow rate range of 200-210 ml/s with substantial prognostic value. Additionally, we observe that a flow rate below 210 ml/m², in association with an aortic valve area ≤1 cm², seems to be predictive for mortality in this group, highlighting the importance of these parameters even within a limited sample size. This observation underscores the importance of incorporating flow rate assessment into risk stratification protocols for optimizing patient management strategies.

It is important to note that further studies are needed to validate the findings and to determine if early intervention will indeed be beneficial in patients with low-gradient severe aortic stenosis and reduced flow rate. Nonetheless, the integration of flow rate as an additional parameter in the evaluation of flow status holds promise for enhancing clinical decision-making processes and improving patient outcomes in this challenging patient population.
